# West Nile Virus: Do We Need its Surveillance and Control Program in Punjab State of India?

**DOI:** 10.4103/0970-0218.66856

**Published:** 2010-04

**Authors:** Tejbir Singh Sandhu, Dalbinder Singh Sidhu, Gursimrat Kaur Sandhu

**Affiliations:** Northwest Mosquito and Vector Control District, Corona, California, USA; 1 Department of Zoology, Punjabi University, Patiala, India; 2 190 Rani Ka Bagh, Amritsar, Punjab, India

West Nile virus (WNv) is an arthropod borne virus of immense public and livestock health importance. Since it’s first isolation in 1937 from West Nile district of Uganda, there is enough literature documenting its isolation, outbreaks, types of hosts, and symptoms.[1–7] WNv is very widely distributed throughout India and has been isolated from mosquitoes,[8–10] bats(11) and humans.[12–14] Presence of WNv-related antibodies in humans was first reported from Mumbai in 1952(15) and confirmed in 1954.(16) Since then, it has been highly prevalent in India. It has been reported in Rajasthan, Maharashtra, Tamil Nadu, Karnataka, Andhra Pradesh, Gujarat, Madhya Pradesh and Orissa and has been associated with human encephalitis cases.[17–21] With respect to other Indian states including Punjab, we were not able to find any published epidemiological study related with this virus in humans except one conducted in Pakistani Punjab where WNv-related antibodies were found in 32.8–38.5% of the sampled human population.(22)

A recent survey conducted by National Institute of Virology (NIV), Pune found significant serological evidence against WNv among horses in and around Pune city of Maharashtra (NIV; unpublished data). Another study published by Post Graduate Institute of Medical Education and Research (PGIMER), Chandigarh in 1999 showed prevalence of JE and WNv infections in pig population in and around Chandigarh.(23)

## What is Needed in Punjab?

Since Northern part of India has vectors as well as reservoir hosts for this virus and its presence has been documented from this region, there is a dire need for setting-up arbovirus surveillance program and suitable testing lab in the state of Punjab. In Punjab, there are seven medical colleges, seven universities (with microbiology or virology departments) and one Regional Disease Diagnostic Laboratory at Jalandhar. With some coordinated effort, an arbovirus surveillance lab can be easily set-up at any one of these institutes. Punjab already has the resources and qualified personnel, which can be appropriately ‘fine-tuned’ with some basic training to establish and run such lab. Infrastructure and field staff of Malaria Control Wing of Health and Animal Husbandry departments can be trained and utilized for fieldwork, e.g. setting and collection of mosquito traps, maintenance and bleeding of sentinel chicken flocks, trapping and bleeding of wild birds, collection of dead birds, etc.

## Need of the Hour

In human medicine, any human encephalitis case that is non-responsive to antibiotics is mostly attributed to diagnosis as ‘viral infection’. These clinical cases are not further investigated for typing of viral infection due to absence of such arbovirus surveillance program and referral lab thus resulting in overlooking of this novel aspect of public health threat. Blood samples from such patients can be shipped to such lab for proper diagnosis and confirmation. Similarly, there is a dire need for screening of all the blood donors for WNv infection to diagnose asymptomatic infections. In Riverside County of California, during the years 2008 and 2009, 22 of 54 (40%) and 2 of 5 (40%), respectively, of the WNv positive human cases were apparently asymptomatic and were detected during screening prior to blood donation (http://www.westnile.ca.gov). Similarly, in veterinary practice, from the professional and personal experience of lead author as field veterinarian in Punjab, he remembers coming across horses with neurological symptoms, which were mostly dubbed as cases of poisoning and therefore routinely went undiagnosed. If such a diagnostic referral lab is established, veterinarians and physicians not only from Punjab but also from the neighboring states will be able to submit blood/serum samples from suspected clinical cases for testing for WNv and other important arboviruses such as dengue fever, outbreaks of which frequently occurs. To conduct a pilot study in support of this projected surveillance program, blood samples can be collected from blood banks situated in different districts of Punjab and can be screened for WNv-related antibodies.

Punjab has a considerable population of horses. According to the 17^th^ Livestock census conducted by Government of India in 2003, the equine population of Punjab was ~34,000.(24) Most of these horses are owned by individuals whose livelihood depend upon the health of these animals. In horses, WNv causes fatal encephalitis(6) with no treatment available yet. But the good news is that there are vaccines available in the world-market for horses and these vaccines provide immunity for 1-5 years. Like foot and mouth disease (FMD) and hemorrhagic septicemia (HS) vaccination programs for cattle, it is recommended that the Punjab government should introduce WNv vaccination program too for its horse population. In addition to above all, a common database of all the surveillance and clinical test results should be established and made available online for free access by physicians, veterinarians, researchers, public and administration. Since this is a disease of public health importance with no specific treatment and human vaccine available till date, rapid exchange of information is very important in updating physicians and veterinarians with clinical data and thus helping each other in forecasting, detecting and understanding the dynamics of this neurological disease.

In the meantime, the only way to fight this disease is by avoiding mosquito bites and controlling mosquito population, which itself is a herculean task if we look at the prevailing living conditions, poverty, open sewage, lack of proper housing among the myriads of other conditions in Punjab. It is virtually next to impossible to avoid mosquito bites in such ecological conditions. The projected surveillance program and diagnostic referral lab will provide excellent basic information on the incidence and prevalence of arboviruses and will assess their public health significance. Use of mosquito repellents during outdoor activities, wearing long sleeved clothing to reduce skin exposure to mosquitoes and reducing mosquito breeding sources from your backyard and neighborhood can drastically reduce the mosquito population. However, the lack of awareness, vast sources of standing water, lack of proper sewage systems and sanitation and lack of adulticiding or larviciding to kill mosquitoes will make this fight against mosquitoes extremely difficult. One point to take into consideration is that there is no treatment and no vaccine available for human use for this fatal neurological disease, so implementation of a basic surveillance program and attempts to control mosquito populations could perhaps also reduce the outbreaks of other vector (mosquito) borne diseases e.g. dengue fever and malaria.
